# The Effect of Carbohydrate Intake on Muscle Hypertrophy: A Systematic Review and Meta-analysis

**DOI:** 10.1007/s40279-025-02341-z

**Published:** 2026-02-19

**Authors:** Menno Henselmans, Fredrik Tonstad Vårvik, Mikel Izquierdo

**Affiliations:** 1 Navarrabiomed, Complejo Hospitalario de Navarra (CHN), Universidad Pública de Navarra (UPNA), Calle de Irunlarrea, 3, 31008 Pamplona, Navarra Spain; 2 Department of Sport Science and Physical Education, University of Agder, Agder, Norway; 3 CIBER of Frailty and Healthy Aging (CIBERFES), Instituto de Salud Carlos III, Madrid, Spain

## Abstract

**Background:**

High-carbohydrate diets are often recommended to enhance resistance training-induced muscle hypertrophy; however, the isolated effect of carbohydrate intake on muscle growth has not been systematically analyzed outside ketogenic diet conditions.

**Objective:**

This meta-analysis aimed to assess whether a higher carbohydrate intake influences muscle hypertrophy under isonitrogenous conditions.

**Methods:**

A systematic search was conducted in the MEDLINE, SPORTDiscus, SciELO, and Google Scholar databases to identify randomized controlled trials comparing higher with lower carbohydrate intakes, whether via supplementation or diet, during resistance training in healthy adults with a measure of muscle size as an outcome. The search was last updated on 26 June, 2025. A random-effects model was used to meta-analyze standardized mean difference (SMD) scores according to Preferred Reporting Items for Systematic Reviews and Meta-Analyses (PRISMA) guidelines. Heterogeneity was assessed using *χ*^2^, *T*^2^, *I*^2^, and prediction intervals. Risk of bias was assessed using the Cochrane risk-of-bias (RoB2) tool. The certainty of evidence was assessed using the Grading of Recommendations Assessment, Development and Evaluation (GRADE) approach. Study quality was assessed using the Assessment of Study Quality and Reporting in Exercise (TESTEX) scale.

**Results:**

Eleven studies met the inclusion criteria. A pooled analysis revealed no significant effect of carbohydrate intake on muscle hypertrophy (SMD = 0.15, *p* = 0.23), with negligible heterogeneity across studies. Sensitivity analyses confirmed the robustness of the findings, and no evidence of publication bias was detected. Subgroup analyses limited to isocaloric trials (SMD = 0.15, *p* = 0.60) and those employing direct imaging of muscle size (e.g., ultrasound) also yielded non-significant results (SMD =  − 0.26 based on only two studies). TESTEX study quality ranged from fair to good with an average score of 9.8 out of 15, but GRADE certainty of evidence was low (2/4) because of imprecision and a moderate risk of bias.

**Conclusions:**

A higher carbohydrate intake may not independently enhance muscle hypertrophy during resistance training, though certainty of evidence is low. Future studies should employ stricter energy intake control and utilize direct morphological assessments to clarify the role of carbohydrate intake independent of total energy balance.

**Clinical Trial Registration:**

PROSPERO CRD42024589461.

**Supplementary Information:**

The online version contains supplementary material available at 10.1007/s40279-025-02341-z.

## Infographic


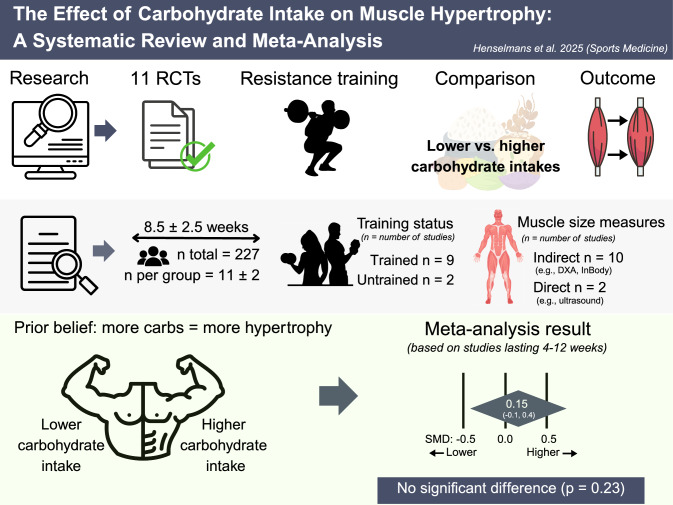


## Key Points

**Table:** 

Our analysis found that carbohydrate intake did not significantly affect muscle hypertrophy.
Carbohydrate intake may be selected based on personal preferences or criteria other than muscle growth, provided that energy and protein requirements are met.

## Background

Resistance training (RT), which relies on a progressive overload to promote gains in muscular strength and hypertrophy, is associated with numerous health benefits [1–4]. Beyond its well-established role in attenuating age-related sarcopenia (a condition affecting 10–27% of adults over 60 years of age characterized by accelerated losses in muscle mass and function) [1], RT improves metabolic health, enhances insulin sensitivity, and contributes to increased longevity [2–4]. Many athletes and recreational trainees also perform RT to build muscle for athletic and aesthetic reasons [5].

Carbohydrate intake is frequently proposed to augment hypertrophic adaptations to RT based on its potential to improve acute performance and support higher chronic training volumes [6, 7]. Higher carbohydrate intakes could also plausibly augment hypertrophy by stimulating insulin and insulin-like growth factor-1 production, although low-carbohydrate diets may not decrease insulin-like growth factor-1 levels when the protein intake is high [8, 9] and carbohydrate intakes and higher insulin levels have not been found to reduce muscle protein breakdown or increase synthesis during high-protein diets [10–12], as protein seems to be insulinogenic enough to make carbohydrates redundant for muscular anabolism. However, the hypothesis that a higher carbohydrate intake increases muscle growth has not been systematically evaluated. A meta-analysis by King et al. [13] showed that pre-exercise carbohydrate ingestion could acutely enhance RT performance; however, the benefits were restricted to fasted-state comparisons, and no clear dose–response relationship was observed. Similarly, a systematic review by Henselmans et al. [14] found no ergogenic effect of carbohydrate supplementation in fed-state RT protocols involving ≤ 10 sets per muscle group, likely owing to limited glycogen depletion.

Indeed, conventional RT protocols typically result in only modest glycogen reductions (≤ 41% [15–26]), falling well below the critical threshold of 250–300 mmol/kg dry weight required to impair neuromuscular function [27]. For instance, Essén-Gustavsson and Tesch reported a 28% reduction in quadriceps glycogen following 20 training sets in bodybuilders [22]. However, Hokken et al. [17] observed that while total quadriceps glycogen depletion was 38% following 12 RT sets, type II muscle fibers experienced greater depletion. Glycogen stores in the lowest quartile declined by 72% (intra-myofibrillar), 60% (inter-myofibrillar), and 62% (subsarcolemmal), with depletion levels in the most-affected fibers approaching thresholds that could impair contractile function. A limitation was the estimation of glycogen depletion based solely on net utilization without accounting for glycogen resynthesis, potentially leading to overestimation. In contrast, Koopman et al. [23] reported lower fiber-specific glycogen depletion of 40% in type IIa and 44% in type IIx fibers after 16 quadriceps RT sets in fasted untrained participants. Furthermore, participants in Hokken et al.’s [17] study, comprising weightlifters and powerlifters, exhibited relatively low baseline glycogen levels (92 mmol/kg wet weight) despite being in a fed state, compared with the higher baseline levels of 120 mmol/kg and 150 mmol/kg reported in strength-trained individuals by Robergs et al. [16] and Haff et al. [19], respectively. An habitual training stimulus may modulate baseline glycogen stores and subsequent depletion during training.

Longitudinal evidence further questions the role of carbohydrates in supporting RT adaptations. A systematic review by Henselmans et al. found no significant differences in strength development in 15 out of 17 long-term studies comparing different carbohydrate intakes; one study favored the higher and one the lower carbohydrate condition [14]. The sole study finding significantly greater strength gains with higher carbohydrate consumption was confounded by unintended caloric restriction and fat loss in the low-carbohydrate group [28]. However, interpretation of these findings is limited because the review did not conduct a quantitative meta-analysis and the included studies varied in methodological quality, with frequent issues such as small sample sizes, inadequate control of total energy intake, and reliance on indirect measures of hypertrophy. Collectively, these data provide a limited mechanistic justification for a direct effect of carbohydrate intake on RT adaptations.

Meta-analyses of ketogenic diets (e.g., Vargas-Molina et al. [29] and Wang et al. [30]) have shown no significant differences in fat-free mass (FFM) increases between high- and low-carbohydrate conditions. However, these analyses were limited by their reliance on indirect assessments of body composition, such as dual-energy X-ray absorptiometry, rather than direct imaging techniques such as magnetic resonance imaging or ultrasound-based assessments of muscle thickness. Additionally, the extraordinary metabolic state of ketosis may restrict the generalizability of these findings to more moderate forms of carbohydrate manipulation [27]. To date, no systematic review or meta-analysis has specifically examined the effect of carbohydrate intake on RT-induced hypertrophy. Therefore, this systematic review and meta-analysis aimed to evaluate the impact of carbohydrate consumption on muscle hypertrophy. Based on the predominance of null findings in the prior literature, we hypothesized that the carbohydrate intake would not significantly influence hypertrophic outcomes.

## Methods

### Study Design

This systematic review and meta-analysis adhered to the guidelines outlined in the Preferred Reporting Items for Systematic Reviews and Meta-Analyses (PRISMA) statement [31]. It was registered in PROSPERO (CRD42024589461) after the methods and search strategy were established but before study screening began.

### Search Strategy

A literature search was conducted using the MEDLINE and SPORTDiscus databases via the EBSCOhost interface and the SciELO database. The search terms included a combination of Medical Subject Headings (MeSH terms) and free text keywords intended to maximize relevant hits based on the authors’ prior publications in this field and pilot testing to ensure the search syntax worked as intended and sentinel publications were included:

“((MH “Carbohydrates” OR DE “CARBOHYDRATES” OR carbohydrate* OR keto* OR (maltodextrin N2 (supplement* OR intake)) OR (glucose N2 (ingestion OR intake OR supplement*))) AND (MH “Resistance Training” OR MH “Weight Lift*” OR DE “RESISTANCE training” OR DE “STRENGTH training” OR isokinetic OR “strength training” OR “resistance training” OR “resistance exercise” OR powerlift* OR weightlift* OR “power lift*” OR CrossFit) AND (MH “Muscle Hypertrophy” OR DE “MUSCULAR hypertrophy” OR DE “MUSCLE growth” OR “muscle growth” OR “muscle volume” OR “muscle size” OR “muscle fiber size” OR “cross-sectional area” OR CSA OR “muscle thickness” OR “body composition” OR “lean body mass” OR “fat-free mass” OR LBM OR FFM)).”Additionally, gray literature (master’s theses, PhD dissertations, and conference abstracts) was searched using Google Scholar via Publish or Perish (Version 8.14.4703). The exact search strategy for each database is included in Appendix A of the Electronic Supplementary Material (ESM). The Google Scholar search was abbreviated because of the 256-character limit. The search was last updated on 26 June, 2025 in Google Scholar and SciELO and on 29 June, 2025 in EBSCOhost. Though the authors are subscribed to virtually all relevant major journals’ e-mail alerts, no formal search alerts were established specifically for this paper in the interim.

### Inclusion Criteria

Randomized controlled trials were included if published online in English and had conditions differing in carbohydrate intake and measured muscle size as an outcome after a RT intervention of at least 6 weeks. Any differences in carbohydrate intake by design, whether via supplementation or diet and regardless of magnitude, were included to maximize the available data for analysis. Studies with differences in protein intake between conditions, whether by design or statistical significance, and studies with differences in supplements other than carbohydrates between conditions were excluded. Eligible measurements of muscle size included any internal measurements of muscle volume or mass, such as muscle thickness or cross-sectional area, as well as estimates of lean body or FFM, using at least a two-compartment model (which estimates fat mass and FFM or more compartments, such as bone). Any measurements that relied on circumference or skinfold calipers were excluded. The study participants had to be metabolically healthy, aged 18–65 years, and free from neuromuscular conditions.

### Study Selection and Data Extraction

MH and FTV screened the titles and abstracts, followed by a review of the complete texts in a blinded manner using Rayyan software. Disagreements were discussed until a consensus was reached. Data from each study were extracted to a spreadsheet (Google Sheets), including (a) citations, (b) study design type, (c) participant characteristics and sample size, (d) daily macronutrient intake, and whether the conditions were isocaloric, along with the estimated energy balance; (e) hypertrophy measurement method; and (f) hypertrophy outcomes (pre and post means and standard deviations [SDs] along with the pre-and post-change scores and pooled baseline SD). In one study [32], WebPlotDigitizer was used to extract data presented only in a graphical format.

### Quality Assessment

Risk of bias was assessed using the Cochrane risk-of-bias (RoB2) tool [33]. The certainty of evidence was assessed using the Grading of Recommendations Assessment, Development and Evaluation (GRADE) approach, following established guidelines [34]. In addition, study quality was assessed using the validated Tool for the Assessment of Study Quality and Reporting in Exercise (TESTEX) scale [35]. For point 5 of the blinded outcome assessment, a point was awarded if it was a full-body laboratory-based scan, even if it was not explicitly specified if the assessor was blinded, as full-body laboratory scans are typically performed by a technician and there is little potential for bias in the first place for automated scans. For point 7 of the intention-to-treat analysis, a point was given if there were no dropouts (and this was recorded); therefore, there was no need for an intention-to-treat analysis. For point 11 of consistent training intensity, a point was assigned if the participants were athletes or strength trainees following their habitual training. The studies were rated as excellent (13–15 points), good (10–12 points), fair (7–9 points), or low quality (< 7 points). RoB2 and GRADE assessments were completed retrospectively during the peer review. All three assessments were performed independently by MH and FTV, followed by discussions of any disagreements until a consensus was reached. MI served as a tiebreaker in the event of an impasse in a disagreement, but this did not occur.

### Statistical Analysis

A random-effects meta-analysis was performed on the standardized mean difference (SMD) scores of hypertrophy outcomes using Comprehensive Meta-Analysis software (version 3; Biostat, Inc., Englewood, NJ, USA). Based on our previous systematic review of this area of research [14], many studies lacked sufficient data to calculate pooled SDs of change scores and contacting all authors to request these data did not result in enough data to base the analysis on pooled SDs of change scores; therefore, the SMDs were calculated as the mean change in condition one minus the mean change in condition two divided by the pooled baseline SD, i.e., Cohen’s *d* [36] in line with conventional practice that allows for an easy comparison of effect sizes across the literature. (Hedges’s *g* was considered because of potentially small sample sizes but was deemed unnecessary and post-hoc indeed resulted in a SMD in the primary analysis that was identical to the first two decimals.) Greene et al. [37] was the only study with a crossover design, and it had sufficient data to calculate the correlation between pre-and post-test scores (*r*); therefore, the SMD variance was calculated based on paired instead of independent samples to account for a within-subject correlation [38]. If multiple effect sizes were present within a single study, they were pooled using inverse variance weighting [39]: this only concerned Wilson et al. [32], for which muscle thickness and lean body mass measurements were pooled. Data are presented as mean ± SD with 95% confidence intervals (CIs). Statistical significance was set at *p* < 0.05. A subgroup analysis was performed for isocaloric versus non-isocaloric comparisons. Condition comparisons were defined as isocaloric if they reported non-significant between-condition differences in energy intake and, in the case of significant between-condition differences in fat mass changes, did not differ by more than 1% in daily body energy balance based on the reported body composition changes using the methods of Hall et al. [40] to account for errors in self-reported dietary data. Another subgroup analysis was performed using only direct local measurements of muscle growth instead of whole-body estimates of lean or FFM. Heterogeneity was assessed using *χ*^2^, *T*^2^, *I*^2^, and prediction intervals [41]. Leave-one-out sensitivity analyses were performed to determine whether any results were conditional on specific study inclusion. Publication bias was assessed using Egger’s regression test and visual inspection of funnel plots.

## Results

### Study Selection and Characteristics

**Fig. 1:**
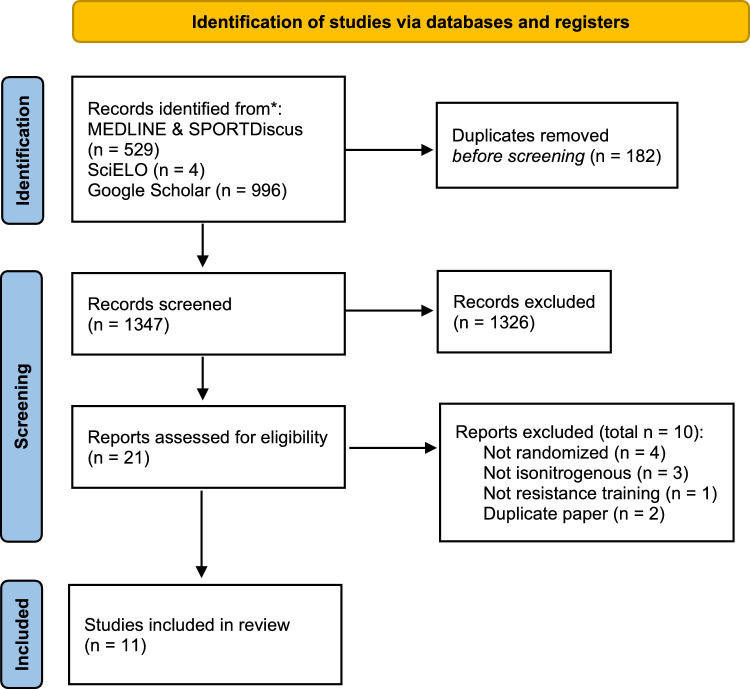
Preferred Reporting Items for Systematic Reviews and Meta-Analyses (PRISMA) flowchart of the study selection process

The systematic literature search yielded 1347 records, of which 21 passed the initial screening, and 11 randomized controlled trials (RCTs) met the inclusion criteria for statistical analysis (see Fig. 1 for the PRISMA flow diagram).

Table 1 provides an overview of the included studies. The average sample size across trials was 20.6 ± 5.6 participants, with an average intervention duration of 8.5 ± 2.3 weeks. Although the study by Krings et al. [42] featured a dietary intervention of 4 weeks, it was retained because of the total intervention duration aligning with our prespecified minimum threshold of 6 weeks. Most studies involved (well-)trained participants, with the exception of Santos et al. [43] and Jabekk et al. [44], which recruited untrained individuals. All trials included male participants [32, 42, 43, 45–48] or a mixed-sex sample [37, 49, 50], except Jabekk et al. [44], who included only women. One study employed a crossover design [37]. Only two studies, Santos et al. [43] and Wilson et al. [32], used direct assessments of muscle size via ultrasound; the remaining studies relied on whole-body FFM as a proxy for muscle hypertrophy.

**Table 1:** Study characteristics

Citation	Design	Sample	Duration (weeks)	Hypertrophy measurement
Sanchez et al. 2024 [49]	Usual diet plus 500 kcal of either a peanut-based or high-carbohydrate snack	19 male and 13 female athletes	10	DXA FFM
Krings et al. 2021 [42]	25% CHO diet plus 70 g of CHO intra-/post-workout vs placebo	18 resistance-trained men	4	Air displacement plethysmography FFM (BodPod)
Santos et al. 2021 [43]	Usual diet plus a post-workout whey shake with or without 50 g of CHO	17 untrained men	8	Ultrasound cross-sectional area
Paoli et al. 2021 [45]	55% CHO Western vs 5% CHO ketogenic diet	19 male bodybuilders	8	BIA FFM (Akern Mod.)
Vidic et al. 2021 [46]	15% CHO vs 5% CHO ketogenic diet	19 resistance-trained middle-aged men	8	BIA FFM (InBody)
Vargas et al. 2018 [47]	55% CHO vs < 10% CHO ketogenic diet	19 resistance-trained men	8	DXA FFM
Gregory et al. 2017 [50]	Ad libitum usual vs ketogenic diet	22 female and 5 male CrossFit trainees	6	DXA FFM
Kysel et al. 2020 [48]	55% CHO balanced vs cyclical ketogenic diet (< 30 g CHO on weekdays, 70% CHO in weekend)	25 resistance-trained men	8	BIA FFM (InBody)
Jabekk et al. 2010 [44]	Regular diet vs ketogenic diet (ad libitum kcal)	16 untrained overweight women	10	DXA FFM
Greene et al. 2018 [37]	Ad libitum usual vs ketogenic diet	7 male and 5 female powerlifters and Olympic weightlifters	12 (in each cross-over condition)	DXA FFM
Wilson et al. 2020 [32]	55% CHO Western vs 5% CHO ketogenic diet with reintroduction CHO in week 11	25 resistance-trained men	11	Ultrasound muscle thickness and DXA FFM

*BIA* bioelectrical impedance analysis, *CHO* carbohydrate, *DXA* dual-energy X-ray absorptiometry, *FFM* fat-free mass

Methodological quality, assessed via the TESTEX scale, ranged from fair (*n* = 3) to good (*n* = 8), with a mean score of 9.8 out of 15; risk of bias was low or moderate, except for high risk for Santos et al. [43]: see Table 2. More details of the TESTEX grading are provided in Appendix B of the ESM and in the Open Science Framework for RoB2.

**Table 2:** Quality and risk-of-bias assessments

Study	Cochrane risk of bias (RoB2)						TESTEX study quality
	Randomization	Deviation	Missing data	Outcome measurement	Reporting	Overall	
Sanchez et al. [49]	Low	Low	Low	Low	Some	Low	11/15
Krings et al. [42]	Some	Some	Low	Low	Some	Some	10/15
Santos et al. [43]	Some	High	High	High	Some	High	8/15
Paoli et al. [45]	Low	Low	Low	Low	Some	Low	11/15
Vidic et al. [46]	Some	Some	Low	Low	Low	Some	10/15
Vargas et al. [47]	Some	Some	Low	Low	Some	Some	10/15
Kysel et al. [48]	Low	Some	Low	Low	Some	Some	11/15
Jabekk et al. [44]	Some	Some	Low	Low	Some	Some	11/15
Greene et al. [37]	Some	Some	Low	Low	Some	Some	7/15
Wilson et al. [32]	Some	Some	Some	Low	Some	Some	9/15
Gregory et al. [50]	Some	Some	Low	Low	Some	Some	10/15
Overall	Some	Some	Low	Low	Some	Some	10/15

### Main Analysis

**Fig. 2:**
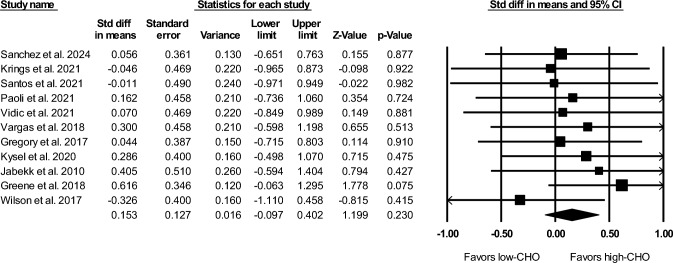
Forest plot of the primary analysis. *CHO* carbohydrate, *CI* confidence interval, *Std diff* standard difference

The primary meta-analysis showed no significant effect of carbohydrate intake on RT-induced hypertrophy (SMD = 0.15; 95% CI − 0.10 to 0.40; *Z* = 1.20; *p* = 0.230). A forest plot of the analysis is shown in Fig. 2. Heterogeneity was non-significant (*Q* = 4.15, *df* = 10, *p* = 0.940); the standard error was 0.127, *I*^2^ value was 0, *τ*^2^ was 0.06, and prediction interval was − 0.19 to 0.49. A fixed-effects model yielded the same point estimate (0.15) for the pooled effect size.

### Subgroup and Sensitivity Analyses

**Fig. 3:**
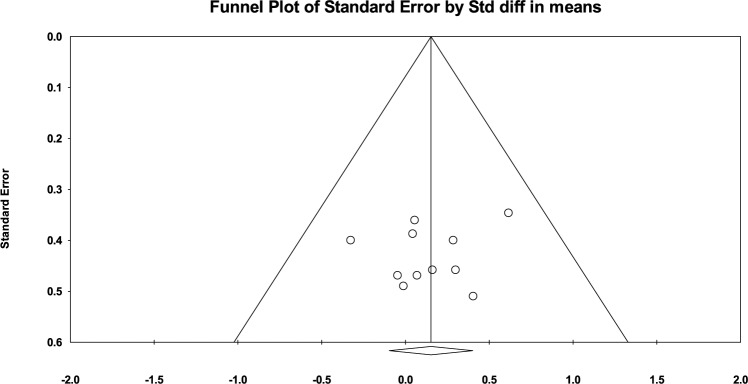
Funnel plot of the included studies. *Std diff* standardized difference

Sensitivity analyses indicated that the main result was robust to the exclusion of any single study, with pooled effect sizes that ranged from 0.080 to 0.206 and corresponding *p*-values between 0.124 and 0.557. The study with the most significant effect on the primary outcomes was that of Greene et al. [37]; its exclusion yielded a reduced pooled effect size of 0.08 (*p* = 0.557). Visual inspection of the funnel plot revealed no evidence of publication bias (Fig. 3). Egger’s regression test supported this, with an intercept of − 0.977 (95% CI − 4.613 to 2.659) and a non-significant *p*-value of 0.558.

**Fig. 4:**
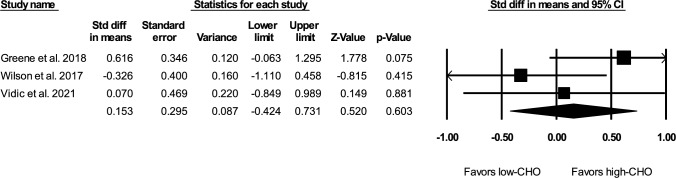
Forest plot of the subgroup analysis of isocaloric studies. *CHO* carbohydrate, *CI* confidence interval, *Std diff* standardized difference

A subgroup analysis of only the studies that met our predefined criteria for isocaloric comparisons found a non-significant effect size of 0.15 (*Z* = 0.520, *p* = 0.603; see Fig. 4) without significant heterogeneity (*Q* = 3.239, *df* = 2, *p* = 0.198, *I*^2^ = 38.259, *τ*^2^ = 0.100). A subgroup analysis of only direct measurements of muscle size found a pooled effect size of − 0.260; as this analysis was limited to two studies [32, 43], no further statistics are reported.

### GRADE Certainty of Evidence

Starting with an initial high certainty of evidence rating because of only including RCTs, we downgraded the certainty level based on the presence of a moderate risk of bias based on the RoB2 assessment. We further downgraded the evidence based on considerable imprecision with the CIs and prediction intervals crossing zero, subgroup analyses potentially changing the direction of effect and sample sizes being small. The studies were reasonably consistent in main effects with non-significant heterogeneity, so no downgrade was applied for inconsistency. The studies included relevant populations, interventions, and outcomes of interest, so no downgraded was applied for indirectness. No downgrade was applied for publication bias because of a lack of evidence thereof. Thus, the final certainty of evidence was rated as low (2/4).

## Discussion

This meta-analysis found no significant effect of carbohydrate intake on RT-induced muscle hypertrophy, and this conclusion remained consistent across the sensitivity analyses, subgroup comparisons, publication bias assessments, as well as all individual studies in the analysis. Our results are consistent with prior meta-analyses examining ketogenic diets, which reported no significant differences in FFM increases between high- and low-carbohydrate dietary regimens [29, 30]. Our results are also consistent with the systematic review of Henselmans et al. [14], which reported no significant difference in long-term strength development between high- and low-carbohydrate conditions in 15 out of 17 studies; one study favored the higher and one the lower carbohydrate condition. Collectively, these findings challenge the prevailing hypothesis that high-carbohydrate consumption enhances muscle growth.

One plausible explanation for the null findings is the limited glycogen depletion typically induced by conventional RT. As previously emphasized by Henselmans et al. [14], training sessions involving ≤ 10 sets per muscle group generally result in glycogen reductions of ≤ 40%—a level generally insufficient to compromise neuromuscular performance, though higher depletion in type II fibers has been reported after higher training volumes. Moreover, mechanical tension is the primary stimulus for muscle hypertrophy, not acute metabolic stress or substrate availability [51].

Another potential reason for the lack of significantly greater muscle growth from higher carbohydrate intakes may be that the between-condition differences in carbohydrate intake were not large enough, as we did filter for any minimum difference thresholds in the search. However, this explanation is hard to reconcile with the similar absence of any significant effect of carbohydrate intake on FFM in prior meta-analyses [29, 30] and measures of muscle growth in any of the included studies, including comparisons with very low-carbohydrate ketogenic conditions [32, 37, 44–48, 50]. Moreover, we performed a post-hoc subgroup analysis without Santos et al. [43] and Krings et al. [42], as these studies conceptually differed in study design by investigating supplemental carbohydrates instead of dietary changes along with different nutrient timing between conditions and lower between-condition differences in total carbohydrate intake than the other interventions. Omitting these studies had a trivial effect on the SMD, which was still non-significant (SMD = 0.18; 95% CI − 0.09 to 0.45; *Z* = 1.32; *p* = 0.184).

Despite the consistency of non-significant effects in our and prior research, the data do not completely rule out a hypertrophic advantage of higher carbohydrate intakes. The pooled SMD was non-significant but favored higher carbohydrate intakes (SMD = 0.15). While the observed heterogeneity was also non-significant (*I*^2^ = 0%, *p* > 0.05) and the 95% prediction interval largely overlaps the CI, true heterogeneity cannot be ruled out and the prediction interval ranged up to 0.49 (from − 0.19), suggesting future studies could report a positive (or negative) effect of carbohydrate intake on muscle growth. Accordingly, the GRADE certainty of evidence was low because of imprecision and a moderate risk of bias, despite TESTEX study quality that ranged from fair to good. If we perform region of practical equivalence testing as per Lakens [52] with bilateral equivalence bounds set by the ‘small’ and ‘moderate’ effect sizes of 0.16 and 0.47, respectively, from Currier et al.’s large-scale meta-analysis of RT interventions [53], the 90% CI of our SMD (− 0.06 to 0.36) falls entirely within the region of practical equivalence of a moderate effect, but the upper end of our CI rejects practical equivalence of a small positive effect. Overall, our data are most consistent with a null effect or a small positive effect of higher carbohydrate intakes on muscle growth.

It is also possible that some individuals respond better to higher carbohydrate diets than others. Ribeiro et al. [54] compared a higher carbohydrate diet (> 5 g/kg/day) to a lower carbohydrate diet and found that while there were no significant differences between the groups in FFM or one repetition maximum strength gains on their three measured exercises, there was a greater proportion of individuals who met the authors’ definition of responsiveness in lean mass and arm curl strength gains. However, this was a non-randomized retrospective analysis of self-selected carbohydrate intakes and the higher carbohydrate group also consumed significantly more total calories and protein, so these confounders limit the attribution of any differences to carbohydrate intake as their cause.

If there is any positive effect of carbohydrate intake on muscle size, it may still not reflect contractile components of lean mass. Previous research has shown that reductions in muscle glycogen and associated water content during low-carbohydrate diets can transiently decrease muscle volume without affecting contractile tissue [55]. This is exemplified by the study of Wilson et al. [32], which uniquely attempted to account for this phenomenon: following 10 weeks on a ketogenic diet, participants who reintroduced carbohydrates at 3.3 g/kg/day during the final week experienced an additional 4.8% increase in FFM—an effect likely attributable to glycogen repletion. In contrast, the control group maintained their carbohydrate intake (3.9 g/kg/day) throughout the study and exhibited no change in FFM during the final week. However, reanalyzing the data without including the 11th week’s carbohydrate reintroduction of Wilson et al. [32] resulted in only a trivial change in the pooled effect size from 0.15 to 0.175, *p* = 0.170. This confounding influence of glycogen and water storage is particularly relevant in studies using whole-body FFM as a surrogate marker for hypertrophy, as these methods may overestimate contractile gains. Intriguingly, our subgroup analysis restricted to direct morphological assessments (i.e., ultrasound-derived muscle thickness) showed a reversal in effect direction, non-significantly favoring a lower carbohydrate intake (SMD =  − 0.260). However, this subgroup comprised only two studies, greatly limiting interpretability. Further research employing imaging-based measures, such as magnetic resonance imaging or ultrasound, is warranted to clarify whether carbohydrate intake influences muscle architecture beyond fluid-related changes.

Furthermore, inadequate control of total energy intake in the included trials may have exaggerated the perceived benefits of higher carbohydrate diets. Low-carbohydrate regimens, particularly ketogenic diets, are well documented to suppress spontaneous energy intake, likely owing to reduced palatability, increased satiety, and dietary monotony [56]. In our dataset, several studies reported no significant between-group differences in self-reported caloric intake. However, their observed body composition changes suggest thermodynamic inconsistencies, unless the lower carbohydrate group experienced a disproportionately greater energy expenditure [37, 44, 50]. A notable example is the study by Greene et al. [37], which met our inclusion criteria for being isocaloric based on self-reported intake and a lack of statistically significant differences in fat mass loss between groups. However, the trial employed an ad libitum design and actual energy balance may have differed between conditions because the high-carbohydrate condition resulted in gains in fat and lean mass, while the low-carbohydrate condition resulted in losses in both. Based on the conditions’ body composition changes and their associated change in bodily energy storage, the higher carbohydrate condition resulted in an approximately 5337 kcal more positive energy balance over the study period. When Greene et al. [37] was excluded from the isocaloric subgroup analysis, the pooled effect size decreased to − 0.159. However, removing Greene et al. [37] left the subgroup with only two studies, which is insufficient for formal statistical analysis, so the effect size change should be regarded as purely hypothesis generating. In light of the hypothesis that the higher carbohydrate groups were on average consuming a higher energy intake, we conducted a post-hoc meta-analysis of changes in fat mass, similar to our analysis of muscle growth, for all studies except Santos et al. [43], which did not report fat mass. There was a non-significant trend for greater fat loss in lower-carbohydrate conditions (SMD = − 0.23; 95% CI − 0.49 to 0.03; *p* = 0.09). Future research employing tightly controlled feeding protocols or objective measures of energy expenditure (e.g., doubly labeled water) is needed to dissociate the independent effects of carbohydrate availability from those of total energy intake on RT-induced hypertrophy.

Nevertheless, in practice, high-carbohydrate diets may be preferred by trainees interested in muscle hypertrophy if this improves adherence to their required energy surplus. Low-carbohydrate diets seem to result in inadvertent energy intake decreases, which may be more suitable for fat loss diets rather than diets aiming to maximize muscle hypertrophy.

Despite conducting a systematic search and meta-analysis based on established methodology, our review had multiple limitations. First, we did not repeat the literature searches from 25 August and 7 September. 2024 after PROSPERO registration on 11 September, so new search hits could have occurred in the interim, although our updated search in 2025 resulted in no new inclusions. Second, while the search strategy aimed to maximize coverage, no explicit systematic framework (e.g., PICO) was used to derive the search terms and sentinel publications were determined based on the authors’ prior publications in this literature, introducing potential for bias. Third, the SciELO search engine yielded anomalous results during both searches, such as illogically higher hit counts when removing an OR operator, so limitations in the database’s search functionality may have restricted retrieval.

### Future Research Recommendations

To overcome the limitations identified in the present review, future studies should prioritize the use of direct morphological assessments, such as ultrasound, magnetic resonance imaging, or muscle biopsy, rather than relying solely on whole-body FFM metrics, which may be more vulnerable to confounding by changes in glycogen and water storage. Incorporating strictly isocaloric designs with emphasis on dietary adherence (e.g., controlled feeding, providing participants with meal plans, prepackaged meals, photo-assisted food logging, and regular dietary supervision) and including periods of carbohydrate refeeding would allow for more accurate isolation of carbohydrate-specific effects. Longer intervention durations (i.e., > 12 weeks) are warranted to determine whether short-term adaptations accurately reflect chronic hypertrophic outcomes. Expanding demographic representation is also warranted; notably, women and older adults remain significantly underrepresented in the RT literature, limiting the external validity of findings. Finally, crossover designs should be considered to enhance statistical power and control for interindividual variability, particularly in studies with small sample sizes. As more data become available, future reviews should consider analyzing specific threshold intakes of carbohydrate and separating supplemental and dietary carbohydrate rather than analyzing higher versus lower intakes across the entire spectrum of carbohydrate intake.

## Conclusions

In summary, the current body of evidence does not support carbohydrate intake as a significant independent determinant of RT-induced muscle hypertrophy. From a practical standpoint, these findings suggest that athletes and clinicians may not need to emphasize carbohydrate intake specifically for hypertrophy, provided that total energy and protein intake requirements are met. However, it should be noted that low-carbohydrate diets may result in reductions in ad libitum energy intake, which may make it more difficult for some individuals to stay in energy surplus. Moreover, the GRADE certainty of evidence was low, so more research is needed to increase confidence in these conclusions.

## Supplementary Information

Below is the link to the electronic supplementary material.

Supplementary file1 (PDF 185 KB)

## Declarations

### Availability of data and material

All data supporting the findings of this analysis are available within the paper, the Appendix, or the Open Science Framework repository: https://osf.io/3y5uh/.
