# Supplementary material for: The Effect of Carbohydrate Intake on Muscle Hypertrophy: A Systematic Review and Meta-analysis
Source: Sports Med. 2026 Feb 19;56(3):691–702. doi: 10.1007/s40279-025-02341-z (PMC13018098; doi:10.1007/s40279-025-02341-z)
Supplement: Supplementary file 1 — Supplementary file1 (PDF 185 KB) [file 40279_2025_2341_MOESM1_ESM.pdf]

Appendix

Title: The Effect of Carbohydrate Intake on Muscle Hypertrophy: A Systematic Review and Meta-Analysis

Journal: Sports Medicine

Menno Henselmans[1], Fredrik Tonstad Vårvik[2], Mikel Izquierdo[1,3]

Corresponding author: Menno Henselmans, info@mennohenselmans.com

[1]Navarrabiomed, Complejo Hospitalario de Navarra (CHN)- Universidad Pública de Navarra (UPNA), Pamplona, Spain

[2]Department of Sport Science and Physical Education, University of Agder, Norway

[3]CIBER of Frailty and Healthy Aging (CIBERFES), Instituto de Salud Carlos III, Madrid, Spain.

Appendix A: literature searches

MEDLINE and SPORTDiscus databases via EBSCOhost

“((MH “Carbohydrates” OR DE "CARBOHYDRATES" OR carbohydrate* OR keto* OR (maltodextrin N2 (supplement* OR intake)) OR (glucose N2 (ingestion OR intake OR supplement*))) AND (MH “Resistance Training” OR MH “Weight Lift*” OR DE "RESISTANCE training" OR DE "STRENGTH training" OR isokinetic OR “strength training” OR “resistance training” OR “resistance exercise” OR powerlift* OR weightlift* OR “power lift*” OR CrossFit) AND (MH “Muscle Hypertrophy” OR DE "MUSCULAR hypertrophy" OR DE "MUSCLE growth" OR “muscle growth” OR “muscle volume” OR “muscle size” OR “muscle fiber size” OR “cross-sectional area” OR CSA OR “muscle thickness” OR “body composition” OR “lean body mass” OR “fat-free mass” OR LBM OR FFM)).”

Google Scholar

Advanced Scholar Search using Publish or Perish (Version 8.14.4703):
(“carbohydrate intake” OR “carbohydrate supplementation” OR “ketogenic diet”) AND ("resistance training" OR weight lifting OR powerlifting OR CrossFit) AND ("muscle hypertrophy" OR "muscle growth" OR "muscle thickness" OR "body composition") AND RCT

Note that the Google Scholar search was abbreviated due to its 256-character limit and RCT was specified to limit irrelevant hits.

SciELO

(Carbohydrate* OR keto*) AND ("resistance training" OR "weight lift*" OR "weightlift*" OR "strength training" OR isokinetic OR "resistance exercise" OR powerlift* OR "power lift*" OR CrossFit) AND ("musc* hypertrophy" OR "muscle growth" OR "muscle volume" OR "muscle size" OR "muscle fiber size" OR "cross-sectional area" OR CSA OR "muscle thickness" OR "body composition" OR "lean body mass" OR "fat-free mass")

Appendix B: study quality overview

	TESTEX point												
**Study**	**1**	**2**	**3**	**4**	**5**	**6**	**7**	**8**	**9**	**11**	**12**	**Total**	**Study quality**
Sanchez et al. 2023	1	1	1	0	1	2	0	2	1	1	1	11	Good
Krings et al. 2020	1	0	1	0	1	2	0	2	1	1	1	10	Good
Santos et al. 2020	1	0	1	1	0	0	0	2	1	1	1	8	Fair
Paoli et al. 2021	1	1	1	1	1	1	1	2	1	1	0	11	Good
Vidic et al. 2021	1	0	1	1	1	2	0	2	1	0	1	10	Good
Vargas et al. 2018	1	0	1	1	1	1	0	2	1	1	1	10	Good
Kysel et al. 2020	1	1	1	1	1	1	1	2	1	1	0	11	Good
Jabekk et al. 2010	1	0	1	0	1	3	0	2	1	1	1	11	Good
Greene et al. 2018	1	0	1	0	1	1	0	1	0	1	1	7	Fair
Wilson et al. 2020	1	0	1	1	1	0	0	2	1	1	1	9	Fair
Gregory et al. 2017	1	0	1	1	1	3	0	2	1	0	0	10	Good
